# The Diagnostic and Predictive Potential of miR-328 in Atrial Fibrillation: Insights from a Spontaneously Hypertensive Rat Model

**DOI:** 10.3390/ijms26073049

**Published:** 2025-03-26

**Authors:** Alkora Ioana Balan, Vasile Bogdan Halaţiu, Emilian Comșulea, Cosmin Constantin Mutu, Dan Alexandru Cozac, Ioana Aspru, Delia Păcurar, Claudia Bănescu, Marcel Perian, Alina Scridon

**Affiliations:** 1 Physiology Department, University of Medicine, Pharmacy, Science and Technology “George Emil Palade”, 540139 Târgu Mureș, Romania; 2 Cardiology Department, Emergency Institute for Cardiovascular Diseases and Transplantation, 540139 Târgu Mureș, Romania; 3 Center for Advanced Medical and Pharmaceutical Research, University of Medicine, Pharmacy, Science and Technology “George Emil Palade”, 540139 Târgu Mureș, Romania; 4 Doctoral School, University of Medicine, Pharmacy, Science and Technology “George Emil Palade”, 540139 Târgu Mureș, Romania; 5 Emergency Clinical County Hospital, 540139 Târgu Mureș, Romania; 6 Genetics Department, University of Medicine, Pharmacy, Science and Technology “George Emil Palade”, 540139 Târgu Mureș, Romania

**Keywords:** animal model, atrial fibrillation, diagnostic biomarkers, microRNAs, predictive biomarkers

## Abstract

Using an atrial fibrillation (AF) model in spontaneously hypertensive rats (SHRs), we aimed to identify circulating miRNAs for AF diagnosis and prediction and to confirm the cardiac origin of these miRNAs. A total of 31 SHRs and 39 Wistar Kyoto (WKY) normotensive controls were randomized into six groups: young, adult, and aging SHR and WKY. Spontaneous AF burden and atrial and circulating levels of 11 miRNAs were quantified. Spontaneous AF was absent in all WKY rats. In the SHRs, AF episodes were observed in two adult animals and in all aging animals (13.6 ± 2.3 episodes/24 h). The atrial levels of five miRNAs were significantly higher in adult and aging SHRs compared to their WKY controls (all *p* < 0.05). Of these, only the circulating levels of miR-328 were significantly higher in the aging SHRs vs. WKYs (*p* < 0.0001). Atrial miR-328 levels in the SHRs increased progressively with age (*p* < 0.001) and correlated with circulating miR-328 levels (r = 0.58; *p* < 0.01). Among aging SHRs, atrial levels of miR-328 strongly correlated with AF burden (r = 0.79; *p* < 0.01). These data suggest that the circulating level of miR-328 could emerge as a promising marker for both AF diagnosis and, if assessed dynamically, for AF prediction.

## 1. Introduction

The prevalence of atrial fibrillation (AF) is steadily increasing with the aging of the population [1]. Often, AF has a paroxysmal and asymptomatic character and, as a result, many patients remain undiagnosed [2]. In the absence of anticoagulant treatment, a large part of these patients remains at risk for serious complications such as stroke [2]. Therefore, diagnostic biomarkers are crucial for early AF detection, risk stratification, and personalized treatment strategies. Arrhythmia prediction would also significantly improve the management of patients prone to AF [3]. Biomarkers that predict arrhythmia onset could play a critical role in intensifying strategies to prevent and/or reverse proarrhythmic remodeling and correct risk factors [4]. Although several biomarkers have been proposed, none of them have demonstrated sufficient accuracy and specificity for reliable AF detection or prediction [4,5]. Approaches that use multiple markers, such as AF risk scores, have also shown limited effectiveness in clinical practice for AF prediction [6].

MicroRNAs (miRNAs) are short, non-coding RNA molecules, crucial for the post-transcriptional modulation of gene expression [7]. In recent decades, clinical and experimental studies have demonstrated the involvement of several miRNAs in AF-associated proarrhythmic atrial remodeling [8]. Previous studies have shown that miR-106b, miR-30, and miR-328 are implicated in calcium handling, a fundamental process for maintaining atrial conduction and electrical stability [9,10]. MiR-150 is recognized for its ability to modulate inflammatory responses within the myocardium, while miR-203 has been associated with regulating mitochondrial function [9,11]. Both inflammation and mitochondrial dysfunction can influence the atrial environment and predispose individuals to arrhythmic conditions [12,13]. Additionally, miR-21, miR-26, and miR-29 are involved in the development of atrial fibrosis, a key factor in structural remodeling that contributes to the substrate for AF [9].

Although the atrial levels of this miRNAs have been proposed as biomarkers for AF diagnosis, quantifying atrial miRNA expression is only feasible in a limited number of patients, such as those undergoing cardiac surgery [8]. Identifying biomarkers for AF prediction in humans is at least as challenging [6]. Due to the large interindividual variability, clinical trials aiming to identify AF predictive biomarkers require large numbers of patients and long-term follow-ups. Animal studies could be used to identify the most promising AF diagnostic and/or predictive biomarkers to be tested in targeted clinical studies, and could thus represent an important step in this regard. The identification of miRNAs associated with AF in experimental animal models can facilitate the discovery of miRNAs that serve as viable biomarkers for arrhythmia diagnosis. Previous research has demonstrated that alterations in miRNA expression are linked to atrial remodeling, and atrial remodeling seems to begin before the arrhythmia onset, thus predicting AF. Consequently, identifying miRNAs that evolve in parallel with atrial remodeling may provide predictive biomarkers for AF.

Accordingly, we aimed to identify circulating miRNAs suitable for AF diagnosis and prediction in an experimental model of AF in spontaneously hypertensive rats (SHRs), and to confirm the cardiac origin of these miRNAs. By identifying those miRNAs that have different expressions in rats manifesting AF and those that remain arrhythmia-free, this study aims to identify those miRNAs that can be used for the diagnosis of AF. On the other hand, by identifying those miRNAs that change progressively with age, this study aims to identify those miRNAs that could be used as predictors of arrhythmia occurrence.

## 2. Results

### 2.1. Systolic Blood Pressure and Mean 24 h Heart Rate in Young, Adult, and Aging Spontaneously Hypertensive and Control Rats

**Figure 1:**
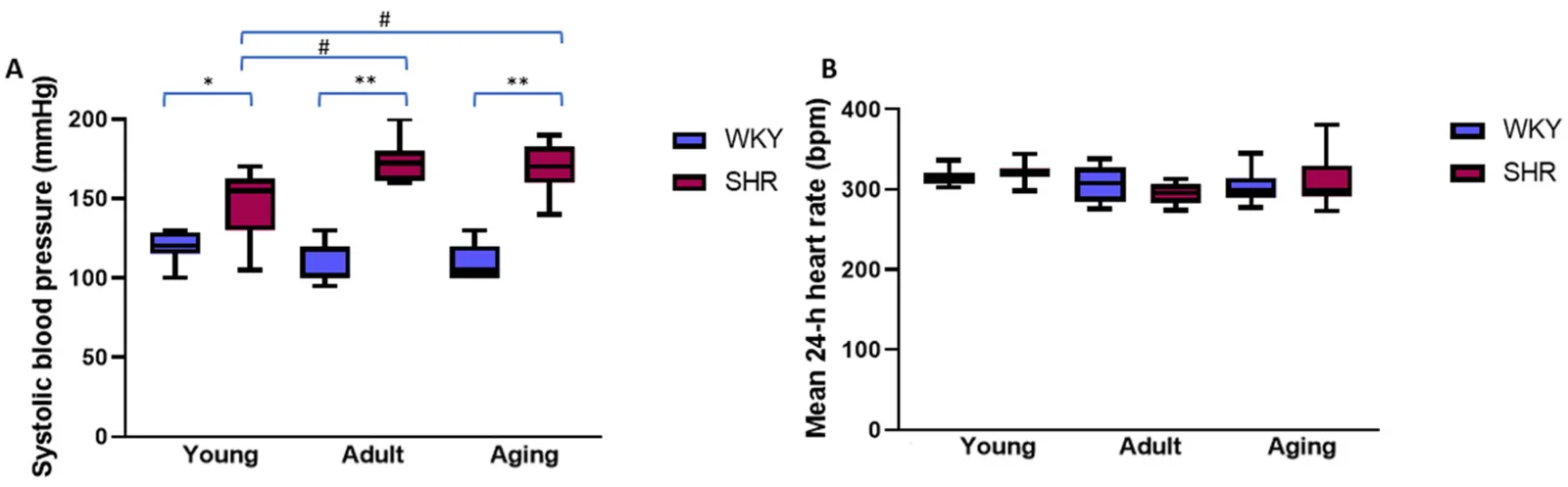
Systolic blood pressure (**A**) and mean 24 h heart rate (**B**) in young, adult, and aging normotensive (WKY) rats and spontaneously hypertensive rats (SHRs). # *p* < 0.01; * *p* < 0.001; ** *p* < 0.0001. Data expressed as medians and interquartile ranges; *p*-values obtained using Mann–Whitney U test.

Systolic blood pressure (Figure 1A) was significantly higher in the SHRs compared to the WKY rats, regardless of their age (all *p* < 0.05). In the SHRs, systolic blood pressure was significantly higher in both the adult (*p* < 0.01) and the aging (*p* < 0.01) rats compared to their young peers, whereas in the WKY rats blood pressure was similar, regardless of the rats’ age (*p* = 0.16). The mean 24 h heart rates (Figure 1B) were not significantly different between young, adult, and aging SHRs and their WKY counterparts (all *p* > 0.05).

### 2.2. Spontaneous Atrial Arrhythmic Burden in Normotensive and Hypertensive Rats

The number of premature atrial contractions (PACs)/24 h was similar between the young SHRs and the WKY rats (18.6 [11.9–25.8] vs. 18.2 [14.0–24.4]; *p* = 0.75), but was significantly higher in both the adult (23.8 [16.4–41.7] vs. 12.2 [6.5–25.7]; *p* = 0.03) and the aging (193.4 [135.2–387.0] vs. 16.8 [7.7–37.8]; *p* < 0.01) SHRs compared to their WKY counterparts. Moreover, in the hypertensive rats, the number of PACs/24 h increased progressively with advancing age (*p* < 0.0001), whereas there was no significant difference in the number of PACs/24 h between the normotensive rat groups, regardless of age (*p* = 0.28).

Regardless of their blood pressure status, none of the young rats presented spontaneous episodes of AF on continuous ECG monitoring. At adult age, two of the hypertensive rats presented spontaneous AF episodes (mean number 0.45 ± 0.18/24 h; mean duration 13.00 ± 12.43 s), whereas arrhythmic episodes were not seen in any of the adult WKY rats. Similarly, none of the aging WKY rats presented spontaneous AF. Meanwhile, all the aging SHRs presented spontaneous AF episodes, with a mean number of 13.68 ± 2.34 AF episodes/24 h and a mean duration of 5.33 ± 1.76 s/AF episode.

### 2.3. Left Atrial and Circulating Expression of Atrial Fibrillation-Related microRNAs in Normotensive and Hypertensive Rats

The atrial expression of the 11 studied miRNAs was not significantly different between young SHRs and young WKY rats (all *p* > 0.05; Table 1). The atrial expression of miR-203 and miR-328 was significantly higher in both the adult and the aging SHRs compared to their WKY counterparts (both *p* < 0.05). In contrast to their WKY controls, in the adult SHRs, the atrial expression of miR-30e was significantly increased (*p* < 0.01), while in the aging rats it was significantly decreased (*p* < 0.001). The atrial expression of miR-150 and miR-99b-5p was significantly lower in both the adult and the aging SHRs compared to their WKY counterparts (all *p* < 0.05), whereas atrial expression of miR-29a was decreased in the aging (*p* = 0.04) but not in the adult (*p* > 0.99) SHRs compared to their WKY controls (Table 1).

**Figure 2:**
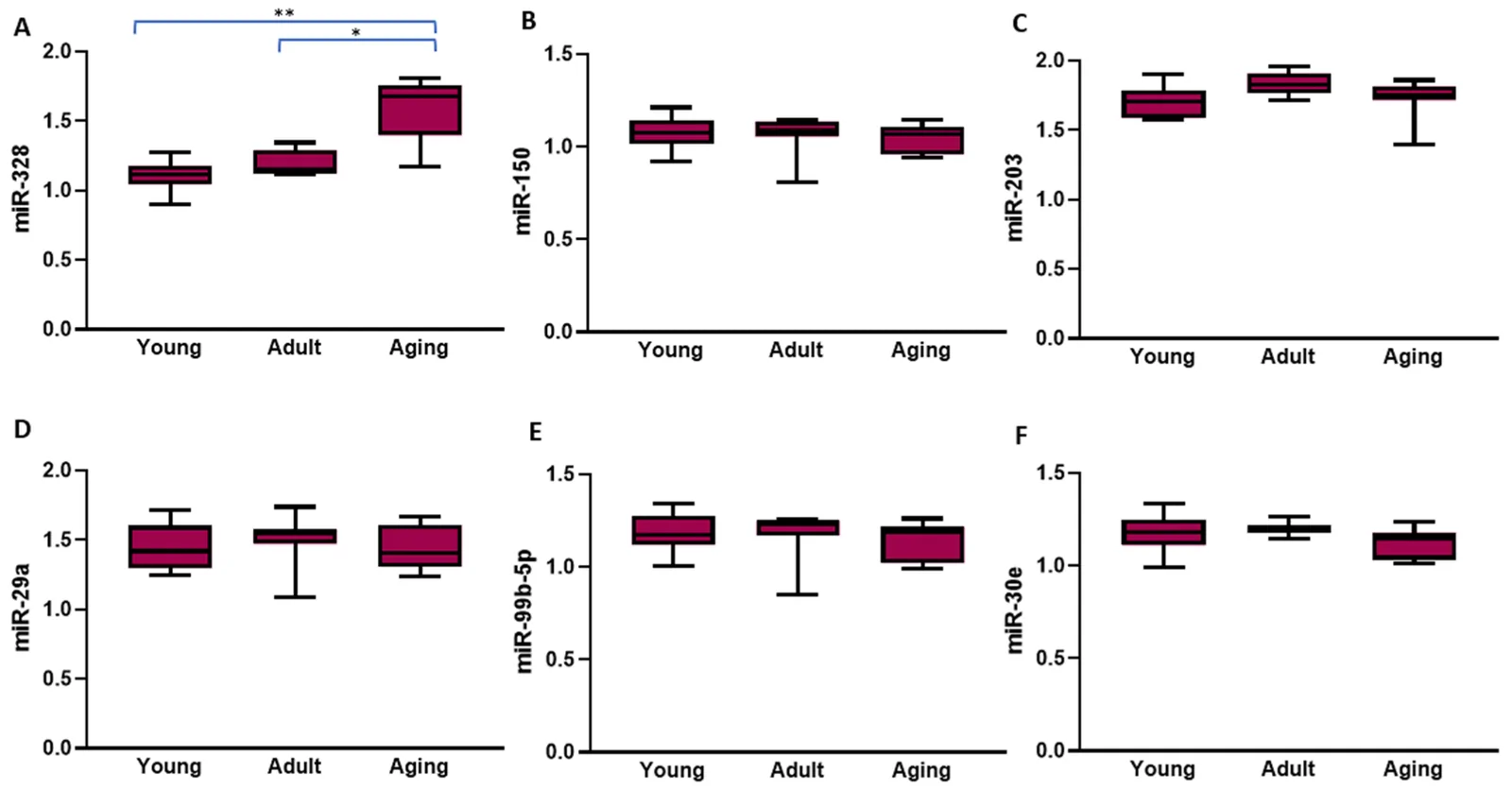
The left atrial levels of the six microRNAs that had significantly different expressions in the spontaneously hypertensive rats compared to their normotensive controls according to age. The values represent microRNA levels normalized with U6 small nuclear RNA levels. The data are expressed as medians and interquartile ranges; the *p*-values presented in this figure were calculated using the Mann–Whitney U test; * *p* < 0.001; ** *p* < 0.0001.

When hypertensive rats of different age groups were compared, atrial expression of miR-328 (Figure 2A) was significantly higher in both the adult and aged rats compared to young rats (both *p* < 0.001). The one-way ANOVA analysis revealed that, among the SHRs, the atrial expression of miR-328 increased progressively with advancing age (*p* < 0.0001) in the context of the presence of arterial hypertension. The expression of all the other miRNAs was similar in all the SHRs, regardless of age (all *p* > 0.05; Figure 2B–F).

Four miRNAs (miR-100-5p, miR-203, miR-29a, and miR-9-5p) were undetectable in the peripheral blood. None of the evaluated circulating miRNAs were significantly different in the young SHRs vs. their normotensive peers (all *p* > 0.05; Table 2). Among the adult rats, the circulating levels of miR-106b (*p* < 0.01) and miR-21 (*p* = 0.02) were significantly higher in the SHRs compared to the WKY rats (Table 2). Higher levels of miR-106b, miR-328, and miR-99-5p were observed in the aging SHRs compared to the aging WKY rats (all *p* < 0.05; Table 2).

**Figure 3:**
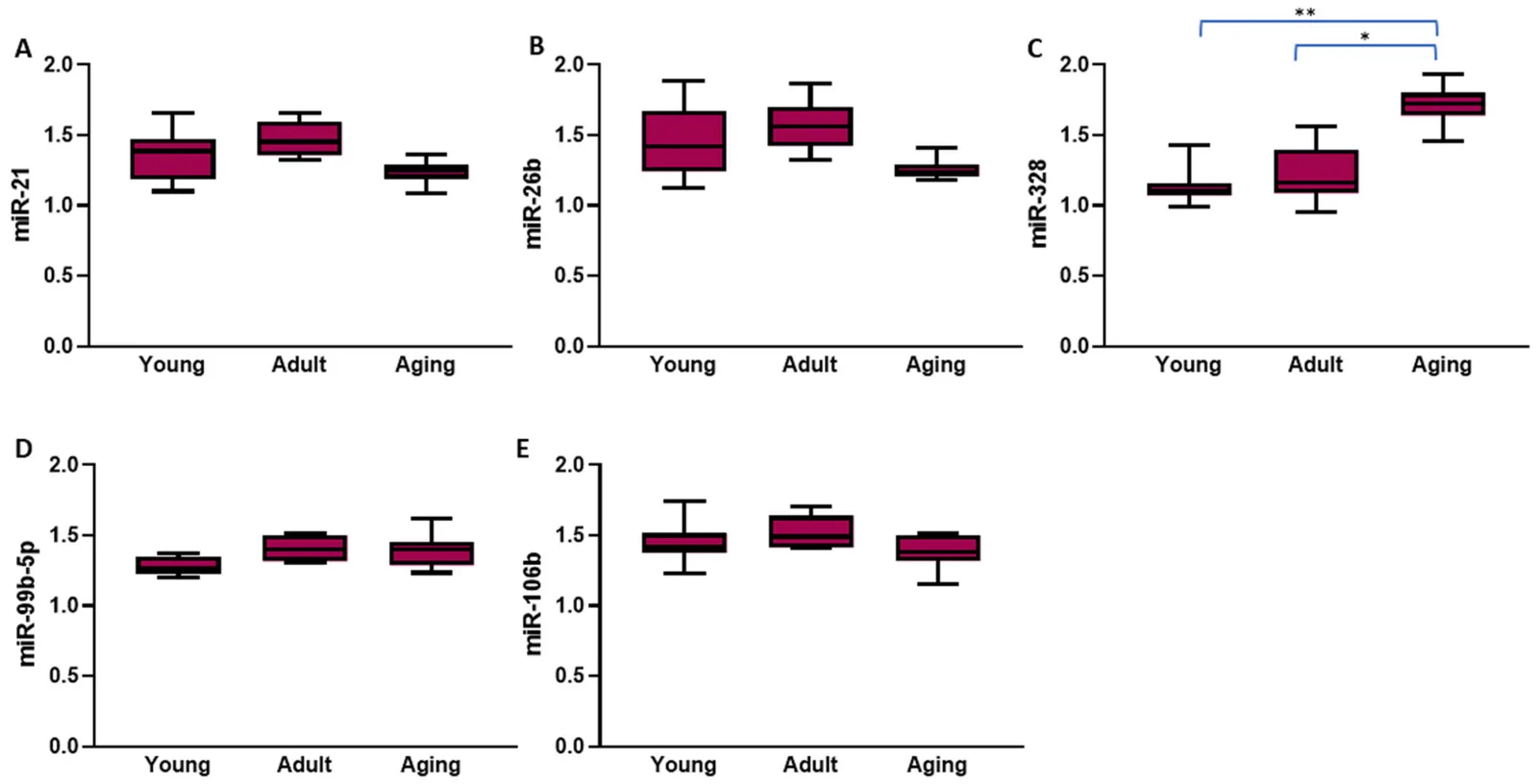
The circulating levels of the five microRNAs that had significantly different expressions in the spontaneously hypertensive rats compared to their normotensive controls according to age. The values represent microRNA levels normalized with U6 small nuclear RNA levels. The data are expressed as medians and interquartile ranges; the *p*-values presented in this figure were calculated using the Mann–Whitney U test; * *p* < 0.01; ** *p* < 0.001.

Among the SHRs, the circulating levels of miR-21 and miR-26b were significantly higher in the adult compared to the aging rats (both *p* < 0.01; Figure 3A,B). Similarly to what was seen in the atrial tissue, in the SHRs, the one-way ANOVA showed that, at least in the hypertensive context, advancing age led to a significant progressive increase in the circulating levels of miR-328 (*p* < 0.001, Figure 3C). No other significant change was seen in the circulating levels of the other evaluated miRNAs with advancing age among the hypertensive rats (all *p* > 0.05; Figure 3D,E).

### 2.4. Atrial-Circulating microRNA Correspondence and Relationship Between miRNAs Levels and Atrial Fibrillation Burden

**Figure 4:**
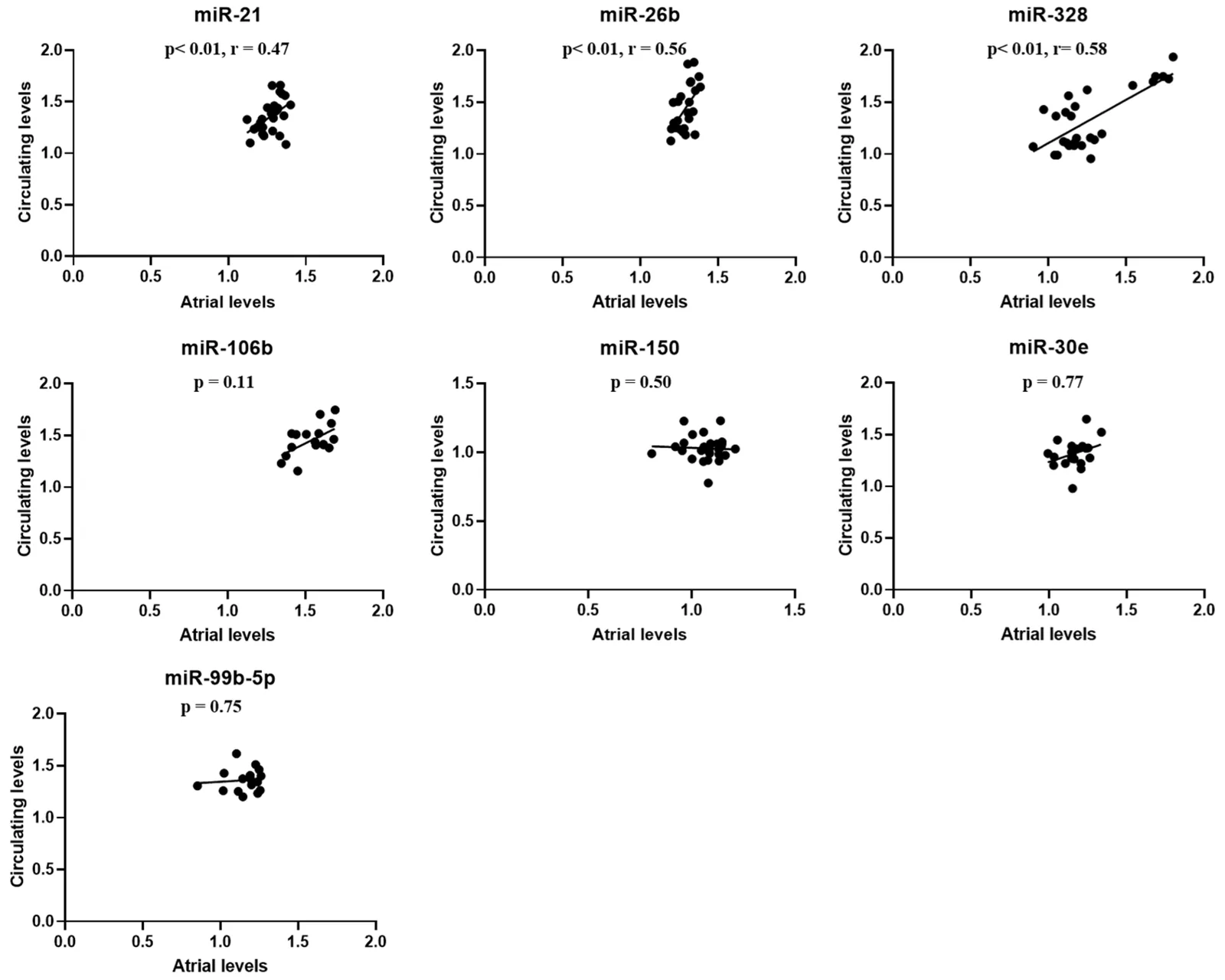
Correlations between atrial and circulating levels of the seven microRNAs that were detectable both in the atrium and in peripheral blood in the spontaneously hypertensive rats. The values represent microRNA levels normalized with U6 small nuclear RNA levels. *p*-values were obtained using the Pearson and Spearman tests, depending on data distribution.

When evaluating the correspondence between the atrial and the circulating levels of the miRNAs that were detectable both in the atrium and in peripheral blood, among the SHRs, significant positive correlations (Figure 4) were ascertained between atrial and circulating levels of miR-21 (r = 0.47; *p* = 0.01), miR-26b (r = 0.56; *p* < 0.01), and miR-328 (r = 0.58; *p* < 0.01). In addition, among the aging SHRs, atrial miR-328 levels significantly positively correlated with the AF burden (r = 0.79; *p* < 0.01). None of the other miRNA levels correlated with the number of AF episodes/24 h in the aging SHRs (all *p* > 0.05).

## 3. Discussion

The main findings of the present study were that: (1) all aging and two adult SHRs presented spontaneous AF, whereas no young SHRs and none of the WKY rats, regardless of their age, presented spontaneous sustained atrial arrhythmic episodes; (2) adult and aging hypertensive rats had higher atrial ectopic activity compared to their normotensive controls; (3) among the SHRs, atrial expression of miR-328 increased progressively with advancing age, (4) significantly positively correlating with circulating miR-328 levels, as well as with (5) spontaneous AF burden.

### 3.1. Aging and Hypertension Create the Optimal Environment for Spontaneous Atrial Fibrillation Occurrence in Rats

The spontaneous occurrence of AF in aging hypertensive rats was first reported in 2012 by Scridon et al. [14]. The present study validates those findings and confirms that the combination of hypertension and advanced age create an optimal environment for spontaneous AF occurrence in rats, in the absence of any artificial factors that could increase AF susceptibility. Both hypertension and advanced age are recognized as major risk factors for AF [5], leading to proarrhythmic atrial structural [15], electrical, autonomic [14,16], and molecular [17,18] remodeling, and AF. As reported in both human patients [19] and rats [14] with spontaneous AF, the number of PACs was also significantly higher in the aging SHRs compared to their normotensive peers, and this increased atrial ectopic activity could have contributed to the increased AF burden observed in the present study in the aging SHRs.

### 3.2. Left Atrial and Circulating Atrial Fibrillation-Related microRNA Changes in Hypertensive Rats

MicroRNAs are small, non-coding RNA molecules that regulate gene expression. This is also applicable to genes that contribute to the structural and electrical remodeling of the atria, such as genes encoding for proteins involved in myocardial fibrosis, hypertrophy, and disturbances in intra-atrial conductivity, which subsequently promote AF occurrence and/or perpetuation [7,19,20,21,22,23]. Elucidating AF-related atrial miRNA changes could thus provide invaluable information regarding the molecular mechanisms involved in AF occurrence, serve as an indicator for the presence and severity of AF-related atrial myopathy, and also facilitate the development of novel, more reliable diagnostic, predictive, and prognostic tools, as well as the identification of novel targets for AF prevention and/or therapy [7,19,20,21,22,23].

Lower left atrial levels of miR-150, miR-99b-5p, miR-29a, and miR-30e and higher levels of miR-203 were observed in the present study in aging SHRs that presented spontaneous AF than in their nonarrhythmic peers. In line with these data, other studies also associated modified atrial levels of miR-150, miR-29, miR-30, and miR-99 with AF [19,20,21,22,23]. Decreased miR-29 levels appear to promote atrial remodeling via the platelet-derived growth factor subunit B (PDGF-B) signaling pathway, while decreased miR-30 levels have been linked with myocardial fibrosis via snail 1 upregulation [24,25]. Although the exact mechanisms by which miR-150, miR-203, and miR-99b are involved in AF are not fully elucidated, they seem to play a role in myocardial inflammation, as suggested by previous studies [26,27,28]. Moreover, all these miRNAs have been implicated in the occurrence of cardiac fibrosis [29,30,31].

As suggested by our and other previously published data, atrial levels of certain miRNAs could thus reflect the degree of atrial remodeling and serve as biomarkers and/or therapeutic targets in AF [7,8]. However, atrial tissue sampling is generally restricted to patients undergoing cardiac surgery or certain invasive cardiac procedures. Meanwhile, circulating miRNAs that accurately reflect atrial miRNA levels could emerge as easily available biomarkers for AF diagnosis and/or prediction.

This does not appear to be the case, however, for miR-150, miR-99b-5p, miR-29a, miR-30e, or miR-203, evaluated in the present study, whose levels were changed only at the atrial level and not in the peripheral blood of aging, arrhythmic SHRs. This finding is not surprising, given that miRNAs generally influence multiple molecular pathways and biological processes and that their expression is rarely restricted to a single type of tissue. MicroRNA-150 [32,33], miR-30 [34,35], and miR-203 [36,37], for instance, are expressed not only in the heart, but also in the colon and bone tissue, and the altered expression of these miRNAs has been linked to colorectal cancer and osteosarcoma. Similarly, miR-29a and miR-99b are also expressed in the liver, lung, and central nervous system [38,39,40,41,42]. In computational studies, although 51 miRNAs were found to be aberrantly expressed in AF, only a small part of them were eligible as biomarkers for AF [43]. Circulating levels of miRNAs such as miR-150, miR-99b-5p, miR-29a, miR-30e, and miR-203 are therefore more likely to reflect systemic changes in gene regulation and do not appear to reliably reflect their atrial expression levels.

The same wide tissue expression and large array of targets for most miRNAs could also explain why miR-106b, 99b-5p, and miR-21 levels were modified in the blood but not in the atria of the SHRs compared to the WKY rats. Although miR-106b and miR-21 are expressed in the atria, they are also expressed in multiple extracardiac tissues [44,45,46]. Circulating miR-21 levels were found to be higher in hypertensive patients than in normotensive subjects [47], and experimental studies showed that miR-21 is also expressed in arterial smooth muscle cells [45]. The altered circulating miR-21 levels seen in our hypertensive rats could thus be the expression of the arterial wall in response to increased pressure values. Moreover, advancing age also seems to influence blood levels of miR-21 [48]. Alterations in circulating miR-106b levels have also been associated with hypertension. Previous studies in spontaneous hypertension models revealed macrophage infiltration within the renal tissues before the onset of hypertension, while macrophage miR-106b-5p secretion has been shown to induce renin production [46]. Together, these results highlight that many of the miRNAs identified to date are in fact non-specific for AF and that identifying miRNAs with altered expression in AF without considering potentially confounding medical conditions and extracardiac expression could lead to confusion regarding AF pathogenesis and the identification of misleading, unreliable AF diagnostic and/or predictive biomarkers. Large-scale, well-controlled studies are therefore needed to confirm the cardiac origin of altered miRNAs identified in the circulating blood in the setting of AF.

To improve the specificity of miRNAs as biomarkers for AF, additional methods to distinguish between cardiac and extracardiac sources of specific miRNAs should be considered in future studies. A promising approach could be the use of exosomes-based miRNA profiling [7,8]. Exosomes are small vesicles that can transport miRNAs from different tissues, and their composition may vary depending on their tissue of origin. By analyzing the miRNAs contained in exosomes derived from atrial versus peripheral blood, we could identify more heart-specific biomarkers and reduce the contribution from extracardiac sources. Also, performing miRNA sequencing on paired samples from both atrial tissue and extracardiac tissues would help identify miRNAs that are predominantly expressed in the heart, and thereby clarify which changes are more likely to be cardiac-specific [7,8]. This method would also help to eliminate potential confounding effects from systemic changes due to hypertension or other conditions.

### 3.3. MicroRNA-328—A Promising Biomarker for Atrial Fibrillation Diagnosis and Prediction

From all the miRNAs tested in the present study, miR-328 emerged as a highly promising biomarker for both the diagnosis and prediction of AF. Both the atrial and circulating levels of miR-328 were significantly higher in the aging, hypertensive, arrhythmic rats compared to their age-matched, normotensive, nonarrhythmic controls. Circulating miR-328 levels closely reflected their atrial levels, and, more importantly, miR-328 levels were strongly correlated with AF burden. The association between increased miR-328 expression and AF has already been reported in previous studies. In dogs, atrial miR-328 levels were upregulated 3.9-fold after AF induction by right atrial tachypacing [49]. The forced expression of miR-328 via adenovirus infection increased AF susceptibility in dogs, while the normalization of miR-328 levels with antagomiR had antiarrhythmic effects. In a recent meta-analysis, circulating levels of miR-328-3p were also found to be significantly higher in AF patients than in nonarrhythmic controls [50]. Our data bring an important addition to these data, demonstrating that the high circulating miR-328 levels associated with AF presence accurately reflect the atrial levels of this miRNA. These findings strongly indicate that the altered circulating levels of miR-328 originate from the atria and are not an expression of extracardiac epiphenomena, thus supporting the role of miR-328 as a promising emerging biomarker for AF diagnosis. Moreover, among the arrhythmic rats, atrial levels of miR-328 strongly positively correlated with the number of spontaneous AF episodes, indicating that miR-328 could emerge as a valuable biomarker not only for the presence but also for the burden of AF.

**Figure 5:**
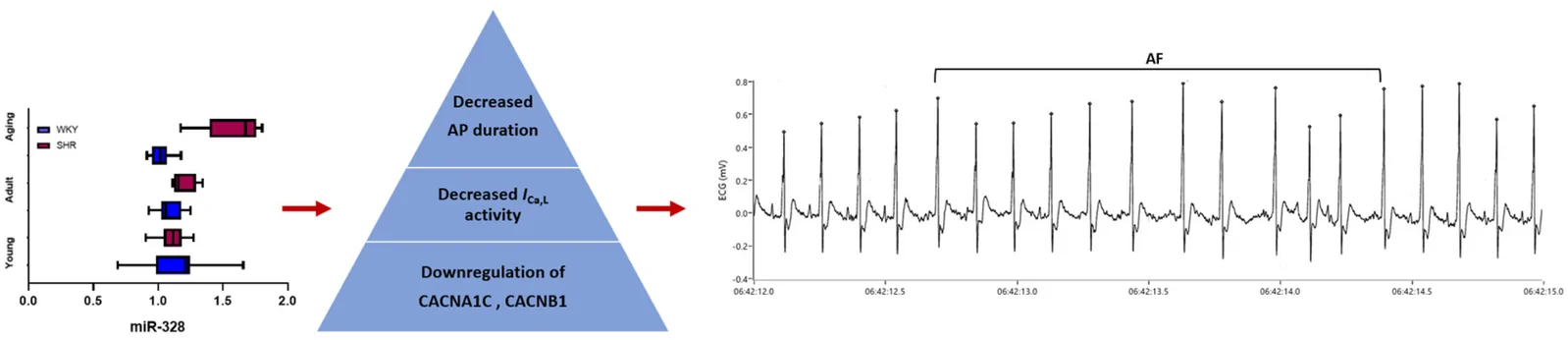
Increased levels of miR-328 in adult and aging SHRs are likely to lead to decreased L-type Ca(2+) current (*I*_Ca,L_) activity and action potential (AP) shortening via the downregulation of CACNA1C and CACNB expression, and, consequently, to the occurrence of premature atrial contractions and atrial fibrillation occurrence. AF—atrial fibrillation; PACs—premature atrial contractions; SHR—spontaneously hypertensive rat; WKY—Wistar Kyoto.

Similarly important was the finding that, among the hypertensive rats, miR-328 expression presented a progressive, age-dependent increase that preceded the onset of arrhythmia. These data suggest that, if assessed in dynamics, miR-328 could emerge as not only a diagnostic but also a predictive AF biomarker, at least in the setting of arterial hypertension. Indeed, previous studies have shown that AF onset is often preceded by proarrhythmic atrial changes [51], and miR-328 has been linked to adverse atrial electrical remodeling, predisposing individuals to AF [49]. Computational analysis identified CACNA1C and CACNB1, responsible for encoding the α1c and β1 subunits of the cardiac L-type Ca(2+) channel, as important targets for miR-328 [49]. Indeed, in experimental models, the forced expression of miR-328 diminished L-type *I*_Ca,L_ activity and decreased action potential duration. A shortened action potential duration can disrupt the coordinated electrical activity in the atria and increase repolarization heterogeneity. These changes create a dispersion of repolarization, promoting the appearance of re-entry circuits and, consequently, AF [5,49]. All these results provide a functional link between miR-328 upregulation and AF (Figure 5).

### 3.4. Potential Limitations

The present study has all the limitations of an experimental study, and its results can obviously not be directly extrapolated to human AF patients. However, the fact that many of the results obtained in this study have already been reported in human patients strongly supports the clinical relevance of these data. Also, in human patients, AF is often a multifactorial disease that occurs in patients with multiple coexisting conditions. The model used in this study reproduces two of the most relevant AF risk factors, aging and hypertension, but does not take into account many other AF risk factors. Thus, these results cannot be extrapolated to other clinically relevant scenarios without validation in other AF-related settings. While a two-way ANOVA with a Bonferroni or Benjamini post hoc test could provide valuable insights into the interactions between age and hypertension status, our study design focused primarily on within-group comparisons at each age rather than on interaction effects. Additionally, considering the sample size and potential deviations from normality, we considered that non-parametric tests ensured a more robust analysis. This limitation leads, of course, to considering the presence of arterial hypertension when interpreting these results.

In this study, a panel of 11 miRNAs previously linked to atrial proarrhythmic remodeling and AF was assessed. The evaluation of additional miRNAs would clearly be of interest. Given the mechanistic data that links miR-328 to AF, studies focusing on the potential of miR-328 as a novel therapeutic target would also be of interest. Although our study provides significant findings regarding the role of miR-328 in AF, a longitudinal approach in additional animal models and, ideally, in human cohorts, would provide a more comprehensive understanding of how miR-328 expression changes over time as AF develops and progresses. Finally, integrating miRNA data with other types of omics data, such as transcriptomic and proteomic studies, could provide a more comprehensive understanding of the molecular pathways involved in AF and help to identify novel therapeutic targets.

## 4. Materials and Methods

### 4.1. Studied Animals

A total of 31 SHRs and 39 Wistar Kyoto (WKY) normotensive controls purchased from Elevage Janvier laboratories (Le Genest Saint Isle, France) were randomized into six groups: young SHR (n = 13) and WKY (n = 16), adult SHR (n = 8) and WKY (n = 12), and aging SHR (n = 10) and WKY (n = 11). The age groups were chosen based on previous studies and the established norms for the SHR model, where age-related physiological and pathophysiological changes, such as the development of hypertension and the onset of atrial remodeling, become more pronounced as rats age. All the animals were housed in a climate-controlled room (21 °C to 23 °C) with a 12 h/12 h light/dark cycle and had free access to standard food and water. All protocols used in the study complied with the International Council for Laboratory Animal Science guidelines (Directive 2010/63/EU) and were approved by the local Ethics Committee and the National Sanitary Veterinary and Food Safety Authority.

### 4.2. Radiotelemetry ECG Monitoring

At 13 weeks of age, young SHRs and WKY rats were implanted with radiotelemetry ECG devices (Data Science International; St. Paul, MN, USA) under isoflurane anesthesia. Adult and aging rats underwent the same procedure at 27 and 47 weeks of age, respectively. The devices were implanted in a subcutaneous pocket in the dorsolateral part of the abdomen, and the two ECG leads were placed subcutaneously in a lead II configuration, as described previously [52]. After 7 days of post-operative recovery, continuous 72 h ECG monitoring was performed in freely moving rats.

**Figure 6:**
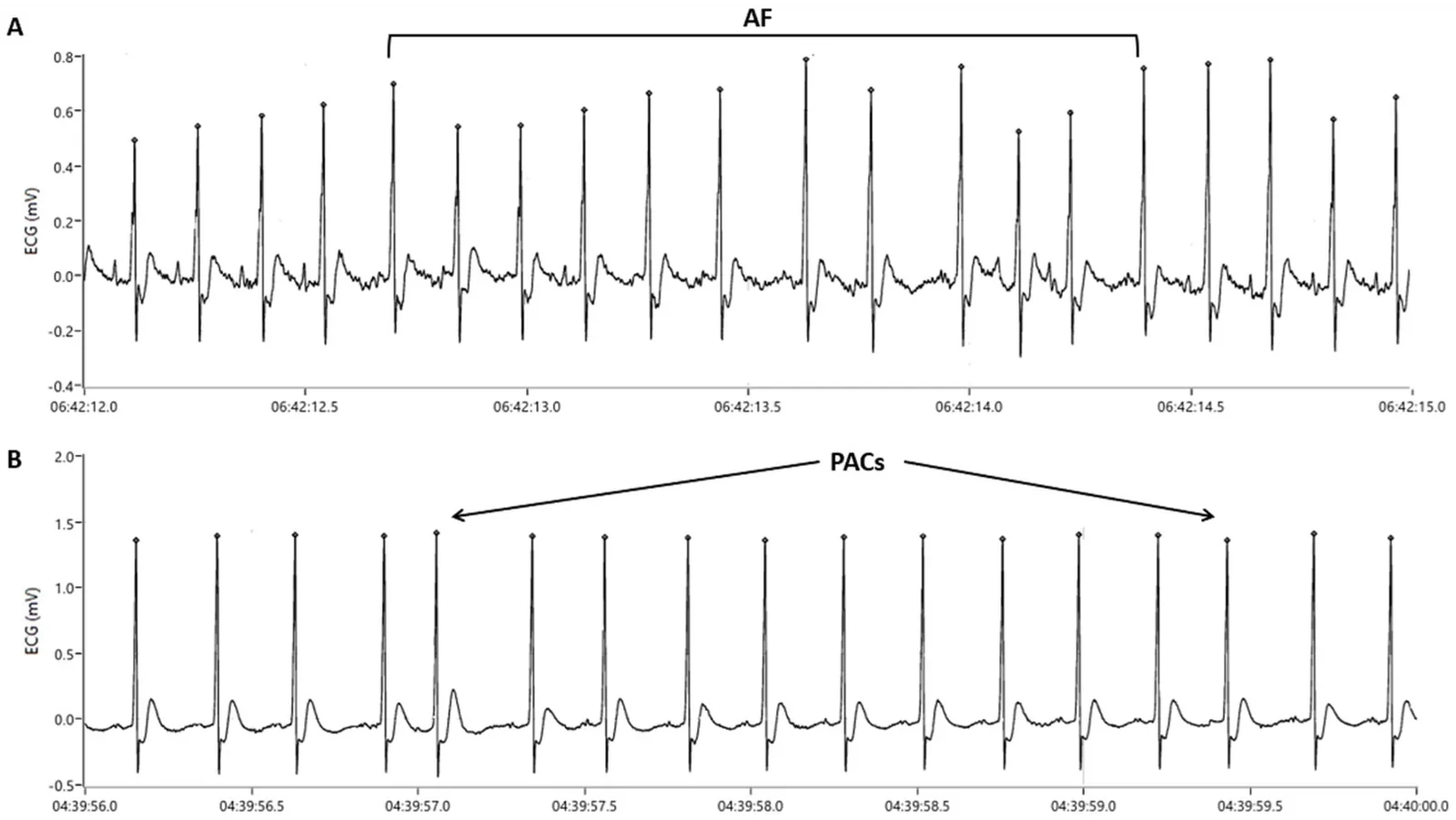
Electrocardiographic tracings showing (**A**) an atrial fibrillation (AF) episode and (**B**) premature atrial contractions (PACs).

Analysis of ECG tracings was performed with a software developed using LabVIEW 2010 (National Instruments; Austin, TX, USA). For each animal, mean daily (24 h) heart rate and arrhythmic burden were evaluated. The number and mean duration of AF episodes and the number of PACs/24 h were assessed and compared between groups. In agreement with previous studies [52], AF was defined as a rapid, irregular rhythm, with narrow QRS complexes and absent P waves, consisting of at least three consecutive QRS complexes (Figure 6A). The duration of an AF episode was measured from the first arrhythmic beat to the first sinus beat that followed the arrhythmic episode. A premature atrial depolarization with different morphology compared to sinus rhythm P waves was defined as a PAC (Figure 6B).

### 4.3. Blood Pressure Measurement

Systolic blood pressure was assessed non-invasively in all animals using the photoplethysmographic method with a PE-300 programmed electro-sphygmomanometer (Narco Bio-Systems; Austin, TX, USA), as described previously [53]. A pneumatic cuff was placed proximally on the rat’s tail, and the photoplethysmography sensor was placed distally, at the level of the caudal artery. Cuff pressure and phototransducer signals were recorded and stored using an acquisition program developed using LabVIEW 8.20. Two measurements were performed for each animal; the values used for comparisons between groups represented the averages of the two values. Blood pressure was measured at 14 weeks of age in the young, 28 weeks of age in the adult, and 48 weeks of age in the elderly rats.

### 4.4. MicroRNA Analysis

At the end of the protocol, all rats underwent isoflurane inhalation anesthesia. The abdominal cavity was opened and blood samples were collected from the abdominal aorta. Circulating levels of 11 target miRNAs (i.e., miR-100-5p, miR-106b, miR-150, miR-203, miR-21, miR-26b, miR-29a, miR-30e, miR-328, miR-9-5p, miR-99b-5p) were evaluated. Subsequently, all animals were euthanized using a terminal dose of phenobarbital sodium (>100 mg/kg of body weight). The hearts were excised, the left atria were sampled, and the left atrial expressions of the 11 miRNAs mentioned above were quantified and compared between the groups.

MicroRNAs from blood and atrial tissue samples were isolated using the MagMAX™ mirVana™ Total RNA Isolation Kit (Thermo Fisher Scientific; Waltham, MA, USA). Reverse transcription was performed using the TaqMan microRNA Reverse Transcription Kit (Thermo Fisher Scientific). MicroRNAs were analyzed with TaqMan assays using Megaplex RT and PreAmp primer pools and were profiled using the OpenArray plate technology on a QuantStudio 12KFlex RT System (Thermo Fisher Scientific). An OpenArray panel was customized to assess the 11 target miRNAs mentioned above. The levels of all the miRNAs were normalized with U6 small nuclear RNA levels and compared between groups.

### 4.5. Statistical Analyses

All statistical analyzes were performed using GraphPad Prism version 8.0.2 software (GraphPad Software; San Diego, CA, USA). All data were subjected to a normality test. Comparisons between groups were performed using the Welch-corrected unpaired t test or the Mann–Whitney U test, as appropriate. Kruskal–Wallis and one-way ANOVA tests were used for multiple comparisons. Correlations were evaluated using the Pearson and Spearman tests, depending on data distribution. Quantitative variables are presented as medians and interquartile ranges or means ± standard errors of the means, as appropriate. Categorical variables are presented as absolute numbers and percentages. The *p*-value was set at 0.05 for statistical significance.

## 5. Conclusions

Recent studies have suggested the potential of certain miRNAs to emerge as novel biomarkers for early AF diagnosis and prognosis. The present study shows that, in a clinically relevant rat model of AF, miR-328 levels are significantly higher in the presence of AF, and demonstrates for the first time that the circulating levels of miR-328 accurately reflect its atrial expression changes, indicating circulating miR-328 as a promising emerging biomarker for the early detection of AF. The progressive, age-dependent increase in miR-328 expression in the hypertensive rats, which preceded the occurrence of arrhythmia, further suggests that dynamic evaluation of miR-328 could emerge as a biomarker to predict arrhythmia occurrence, at least in selected categories of patients. Awaiting confirmation in clinical settings, the miRNA changes observed in this study could serve as a basis for future targeted clinical studies aimed at developing more accurate diagnostic and predictive AF tests and personalized intervention strategies for patients with AF.

## Figures and Tables

**Table 1:** Atrial microRNA (miR) expression in young, adult, and aging spontaneously hypertensive rats (SHR) and normotensive controls (WKY).

MicroRNAs	Young		*p*-Value	Adult		*p*-Value	Aging		*p*-Value
	WKY (n = 16)	SHR (n = 13)		WKY (n = 12)	SHR (n = 8)		WKY (n = 11)	SHR (n = 10)	
**miR-100-5p**	1.27 [1.19–1.35]	1.22 [1.19–1.28]	0.83	1.26 [1.25–1.31]	1.26 [1.22–1.28]	0.52	1.25 [1.21–1.27]	1.24 [1.19–1.28]	0.64
**miR-106b**	1.59 [1.39–1.64]	1.56 [1.41–1.67]	0.94	1.55 [1.50–1.56]	1.59 [1.51–1.61]	0.28	1.55 [1.54–1.61]	1.47 [1.41–1.63]	0.19
**miR-150**	1.13 [0.99–1.17-	1.07 [1.01–1.14]	0.73	1.14 [1.11–1.16]	1.08 [1.05–1.13]	0.03	1.16 [1.14–1.17]	1.07 [0.95–1.10]	<0.01
**miR-203**	1.74 [1.61–1.86]	1.70 [1.58–1.78]	0.56	1.62 [1.56–1.68]	1.82 [1.76–1.91]	<0.01	1.59 [1.54–1.68]	1.75 [1.71–1.81]	<0.01
**miR-21**	1.30 [1.10–1.42]	1.28 [1.21–1.31]	0.77	1.30 [1.27–1.32]	1.30 [1.26–1.33]	0.58	1.26 [1.25–1.29]	1.23 [1.20–1.34]	0.52
**miR-26b**	1.28 [1.26–1.37]	1.27 [1.22–1.33]	0.58	1.27 [1.23–1.34]	1.31 [1.25–1.32]	0.47	1.24 [1.21–1.30]	1.27 [1.23–1.31]	0.65
**miR-29a**	1.55 [1.22–1.59]	1.41 [1.29–1.60]	0.85	1.54 [1.50–1.58]	1.54 [1.47–1.57]	>0.99	1.59 [1.51–1.62]	1.40 [1.30–1.60]	0.04
**miR-30e**	1.25 [1.06–1.29]	1.18 [1.11–1.24]	0.8	1.07 [0.98–1.11]	1.20 [1.17–1.21]	<0.01	1.27 [1.26–1.29]	1.14 [1.03–1.17]	<0.001
**miR-328**	1.20 [0.98–1.24]	1.11 [1.04–1.17]	0.64	1.05 [1.02–1.17]	1.15 [1.12–1.29]	0.01	1.01 [0.95–1.06]	1.67 [1.39–1.75]	<0.0001
**miR-9-5p**	1.55 [1.45–1.67]	1.51 [1.46–1.57]	0.39	1.50 [1.48–1.55]	1.56 [1.52–1.58]	0.13	1.50 [1.45–1.54]	1.48 [1.41–1.56]	0.74
**miR-99-5p**	1.28 [1.06–1.33]	1.17 [1.11–1.27]	0.42	1.27 [1.25–1.28]	1.23 [1.17–1.25]	0.01	1.26 [1.22–1.28]	1.19 [1.02–1.21]	<0.01

MicroRNA values presented in the table represent microRNA levels normalized with U6 small nuclear RNA levels. Data expressed as medians and interquartile ranges; *p*-values obtained using Mann–Whitney U test.

**Table 2:** Circulating microRNA (miR) levels in young, adult, and aging spontaneously hypertensive rats (SHRs) and normotensive controls (WKYs).

MicroRNAs	Young		*p*-Value	Adult		*p*-Value	Aging		*p*-Value
	WKY (n = 16)	SHR (n = 13)		WKY (n = 12)	SHR (n = 8)		WKY (n = 11)	SHR (n = 10)	
**miR-106b**	1.37 [1.29–1.56]	1.42 [1.37–1.51]	0.57	1.26 [1.14–1.32]	1.49 [1.41–1.63]	<0.01	1.22 [1.12–1.33]	1.38 [1.31–1.5]	0.01
**miR-150**	1.03 [0.89–1.09]	1.03 [0.97–1.07]	0.47	0.96 [0.74–1.06]	1.02 [0.99–1.06]	0.06	0.84 [0.78–0.99]	1.03 [0.97–1.06]	0.09
**miR-21**	1.31 [1.23–1.40]	1.38 [1.18–1.46]	0.57	1.33 [1.18–1.44]	1.45 [1.35–1.59]	0.02	1.20 [1.15–1.32]	1.25 [1.19–1.28]	0.78
**miR-26b**	1.32 [1.27–1.51]	1.41 [1.24–1.67]	0.44	1.35 [1.27–1.63]	1.55 [1.42–1.69]	0.10	1.29 [1.16–1.48]	1.24 [1.20–1.29]	0.27
**miR-30e**	1.27 [1.18–1.33]	1.34 [1.23–1.48]	0.21	1.20 [1.07–1.35]	1.31 [1.27–1.38]	0.07	1.18 [1.05–1.29]	1.31 [1.18–1.40]	0.09
**miR-328**	1.17 [1.04–1.25]	1.11 [1.07–1.15]	0.34	1.19 [0.95–1.29]	1.16 [1.08–1.39]	0.40	1.10 [1.05–1.33]	1.72 [1.64–1.80]	<0.0001
**miR-99-5p**	1.29 [1.04–1.41]	1.26 [1.22–1.34]	>0.99	1.19 [1.08–1.41]	1.40 [1.31–1.49]	0.19	0.99 [0.93–1.25]	1.40 [1.28–1.45]	0.01

MicroRNA values presented represent microRNA levels normalized with U6 small nuclear RNA levels. Data expressed as medians and interquartile ranges; *p*-values obtained using Mann–Whitney U test.

## Data Availability

The datasets can be obtained from the corresponding author on request.

## Abbreviations

The following abbreviations are used in this manuscript:

AF	atrial fibrillation
miR	microRNA
miRNAs	microRNAs
PACs	premature atrial contractions
PDGF-B	platelet-derived growth factor subunit B
SHR	spontaneously hypertensive rat
WKY	Wistar Kyoto
