# Correction: Balan et al. The Diagnostic and Predictive Potential of miR-328 in Atrial Fibrillation: Insights from a Spontaneously Hypertensive Rat Model. *Int. J. Mol. Sci.* 2025, *26*, 3049

**DOI:** 10.3390/ijms27177679

**Published:** 2026-08-27

**Authors:** Alkora Ioana Balan, Vasile Bogdan Halaţiu, Emilian Comșulea, Cosmin Constantin Mutu, Dan Alexandru Cozac, Ioana Aspru, Delia Păcurar, Claudia Bănescu, Marcel Perian, Alina Scridon

**Affiliations:** 1 Physiology Department, University of Medicine, Pharmacy, Science and Technology “George Emil Palade”, 540139 Târgu Mureș, Romania; 2 Cardiology Department, Emergency Institute for Cardiovascular Diseases and Transplantation, 540139 Târgu Mureș, Romania; 3 Center for Advanced Medical and Pharmaceutical Research, University of Medicine, Pharmacy, Science and Technology “George Emil Palade”, 540139 Târgu Mureș, Romania; 4 Doctoral School, University of Medicine, Pharmacy, Science and Technology “George Emil Palade”, 540139 Târgu Mureș, Romania; 5 Emergency Clinical County Hospital, 540139 Târgu Mureș, Romania; 6 Genetics Department, University of Medicine, Pharmacy, Science and Technology “George Emil Palade”, 540139 Târgu Mureș, Romania

## Reference Correction

There was a citation error in the original publication [1]. Reference [34], cited in the published manuscript, was retracted by the publisher.

To uphold the highest standards of academic integrity and ensure the accuracy of the scientific record, the authors replaced the retracted reference with the following:

34. Zhang, Q.; Yu, L.; Qin, D.; Huang, R.; Jiang, X.; Zou, C.; Tang, Q.; Chen, Y.; Wang, G.; Wang, X.; et al. Role of microRNA-30c targeting ADAM19 in colorectal cancer. *PLoS ONE* **2015**, *10*, e0120698.

The authors state that the scientific conclusions are unaffected. This correction was approved by the Academic Editor. The original publication has also been updated.
